# Study determinants of increased Z-Score of Body Mass Index in preschool-age children

**DOI:** 10.1186/s13104-020-05026-0

**Published:** 2020-03-30

**Authors:** Fathi M. El-Gamal, Rawan Babader, Maha Al-Shaikh, Amani Al-Harbi, Jumana Al-Kaf, Wafa Al-Kaf

**Affiliations:** 1 Department of Family Medicine, Ibn Sina National College for Medical Studies, Al Mahjer Street, P.O. Box 31906, Jeddah, 21418 Kingdom of Saudi Arabia; 2 Ibn Sina National College for Medical Studies, Jeddah, Kingdom of Saudi Arabia

**Keywords:** BMI Z score, Stunting, Allergic disorders, Preschool children, Obesity

## Abstract

**Objective:**

To determine the association between socioeconomic level, gender, stunting and other characteristics with the presence of overweight/obesity in the preschool children.

**Result:**

BMI/Age Z score > + 2 SD was found in 19.5% of the children. It was more common among the children from areas with high socio-economic level (OR: 2.43; 95% CI 1.54, 3.84, and p < .000). Obesity was higher among the males (OR 1.76; 95% CI 1.09, 2.8, and p < .02) compared to females. The increased duration of breast feeding, was significantly associated with increased BMI/Age Z-score (b = .027, p < .004). Decreased age of the child was significantly associated with increased BMI/Age Z-score (b = −  .013, p < .004). The children with stunted growth were 6.7 times fold likely to have BMI/Age Z Score > + 2 SD compared to the normal children (OR 6.73; 95% CI 3.79, 10.80, and p < .000), after allowing for other factors. No significant association was found between allergic disorders and BMI/Age Z score > + 2 SD. Thus male gender, high socioeconomic condition, increased duration of breast feeding and stunting were significantly associated with overweight/obesity in preschool children.

## Introduction

Pediatric overweight and obesity have increased worldwide [1]. It is linked with increased risk of long-term ailments in adulthood [2]. Prevalence of overweigh and Obesity in developed countries is higher compared with developing regions of the world [3]. Overweight tended to be more common among girls, particularly in developing countries [4]. Associations between asthma and high BMI have been observed in studies of children and adults [5, 6]. This study focused on exploring the possible associations between socio-demographic, personal and clinical characteristics with overweight/obesity in preschool children.

## Main text

### Methods

This is a cross-sectional study, where a convenient sample of preschool children who visited the outpatient clinics with their relatives, but not as patients, in two hospitals during 2 month-period was selected. One hospital was in the North of Jeddah, which serves a well off community with high socioeconomic level, while the other hospital was in the South of Jeddah city, which serves a community with low socioeconomic level. The total number of children examined was 748; this number was greater than the required minimum number for such a study (220 children) [7]. Data was collected from children’s mothers after taking an informed written consent. Data was collected through: (1) questionnaire: which provided information about personal and socio-demographic characteristics, feeding patterns, and clinical characteristics of the child; (2) anthropometry: weights and lengths/heights of the children and their mothers according to standard procedures. Anthropometric analysis: The variables age, sex, weight and height were used. These measurements were used to provide the following indices: weigh-for-age, height-for-age, weight-for-height, and BMI for age. The indices generated were compared to standard reference values of WHO to obtain the corresponding Z-scores [8]. We used Z-scores to determine the nutritional status of the children. A child whose height-for-age Z-score was < − 2, from the median value of the reference population, was considered as stunted. The children with BMI/Age Z-score > + 2 Z to + 3 Z, from the median value of the reference population, were classified as overweight; and if it was > + 3 Z, obese.

3-ISAAC core questionnaire on asthma and allergy: It is used to diagnose bronchial asthma, allergic rhinitis and atopic eczema [9].

Data analysis and statistical tests: The data was analyzed using the Statistical Package for Social Sciences (IBM SPSS, version 23, Armonk, NY: IBM Corp.). The Multi-nominal Logistic regression method was used where BMI/Age Z score > + 2 SD was used as the dependent dichotomous variable and other variables were used as the independent dichotomous variables, where Odds ratios, 95% confidence interval (95% CI), and p values were calculated. The Linear Multiple Regression Analysis was used to study the continuous variables that could significantly predict the BMI/Age Z Score. The level of significance was .05.

### Results

The males were 357 (47.7%), and 391 were females (52.3%). The BMI/age Z score > + 2 SD was found in 146 children (19.6%). Among the studied children 64 were overweight (8.6%), while 82 were obese (11.0%). The Mean age of the children who have BMI/Age Z score > + 2 SD was 29.19 months (SD 19.92), compared to 34.69 months (SD 20.79) for children who have BMI/Age Z score ≤ 2 SD (t-test = 2.887, p < .004). The BMI/Age Z Score > + 2 SD was significantly more encountered among children in the Northern region compared to children in the Southern region of Jeddah (OR: 2.43; 95% CI 1.54, 3.84, and p < .000). The BMI/age Z score > 2 SD was found in 23.8% of the males, while it was recorded in 16% of the females. The BMI/Age Z score > + 2 SD was significantly more common among the males compared to the females (OR 1.76; 95% CI 1.09, 2.8, and p < .02). However, the other socio-demographic factors like parents educational level, their occupation, or their monthly income were irrelevant to BMI Z score > + 2 Z among the studied children. The types of food administered to the children were irrelevant to BMI/Age Z scores > + 2 SD (Table 1). The younger children have significantly higher values of BMI/Age Z-scores compared with the older ones (b = − .013, p < .004). The family size, and the rank of the child among his siblings were, also, not significant determinants of the child BMI/Age Z score. The increased duration of breast feeding in infancy, was significantly associated with the increase in BMI Z-score (b = .027, p < .004). The children with stunted growth were 6.7 times fold more likely to have BMI/age Z Score > + 2 SD compared to the normal children (OR 6.73; 95% CI 3.79, 10.80, and p < .000), after controlling for the other factors. The BMI/Age Z score > + 2 in the children was independent of the BMI for their mothers (Table 2), and was, also, independent of having asthma, or allergic rhinitis, or eczema or other infections (Table 3).

**Table 1:** Logistic regression of the different independent variables on BMI/Age Z Score over 2 SD

Independent variables	B	Sig.	Exp(B)	95% Confidence Interval for Exp(B)	
				lower bound	Upper bound
Intercept	1.765	.001			
Area of residence	.890	.000	2.434	1.543	3.841
Nationality	− .089	.664	.915	.613	1.366
Gender	.570	.022	1.768	1.086	2.879
Educational level of the father	.157	.467	1.170	.766	1.786
Monthly income	.104	.672	1.109	.686	1.793
Educational level of the mother	− .049	.824	.953	.621	1.460
Occupational of the father	.053	.814	1.054	.680	1.633
Occupational of the mother	.110	.657	1.116	.688	1.811
Monthly income	.104	.672	1.109	.686	1.793
Gestational period	− .208	.543	.812	.415	1.588
Main feeding in infancy	− .034	.868	.966	.645	1.448
Eat food with preservatives, daily	− .176	.461	.839	.526	1.338
Eat sweet food daily	− .097	.668	.907	.582	1.414
Drink milk, daily	− .079	.752	.924	.567	1.507
Eat fruits and vegetables, daily	− .344	.191	.709	.423	1.187
Treatment for anemia	.030	.947	1.031	.425	2.497

**Table 2:** Multiple regression of independent variables and BMI/Age Z-Score

	Unstandardized coefficients		Standardized coefficients	t	Sig.
	B	Std. error	Beta		
(Constant)	.328	.442		.742	.458
Age (Months)	− .013	.004	− .139	− 2.915	.004
Number of children in the family	− .185	.132	− .137	− 1.405	.161
Rank of the child among his siblings	.122	.132	.089	.923	.357
Duration of main feeding in infancy	.027	.010	.129	2.872	.004
BMI of the mother	.000	.012	.000	.010	.992

**Table 3:** Logistic regression between BMI/Age Z Score over 2 SD and allergic disorders and stunting

Independent variables	B	Sig.	Exp(B)	95% Confidence Interval for Exp(B)	
				Lower Bound	Upper Bound
Intercept	− .117	.781			
Stunting growth	1.900	.000	6.688	4.438	10.079
ISAAC diagnosed asthma	.129	.588	1.138	.713	1.817
ISAAC diagnosed rhinitis	.377	.153	1.458	.869	2.448
ISAAC diagnosed eczema	− .558	.245	.572	.224	1.465
Repeated respiratory tract infection	− .331	.291	.719	.389	1.328
Family history of allergy	.212	.312	1.236	.820	1.863

### Discussion

Early years of life is a critical period for evaluating the onset of obesity and application of control strategies; as increased BMI in childhood is linked to obesity and ill health in later years [10]. The weight of children naturally fluctuates during growth, and so assessment of BMI in pediatric age groups necessitates the availability of reference charts which consider gender and age. A child with BMI for age and gender Z-score that is > 2 SD from the median of the reference population is classified as overweight, and if it is > 3 SD is considered obese [8]. The main objective of the present study was to assess the prevalence and determinants of BMI/age Z score > 2 SD among preschool children in Jeddah city KSA, using the WHO Child Growth Standards. Based on data collected from sample of the child population in Jeddah city KSA it was found that approximately 20% of preschool children have BMI/age Z score > 2 SD. This is contradicting to a previous study [11] who revealed that the overall prevalence of overweight in preschool children in developing countries was low (3.3%) [12]. Findings of the present study, also, are comparable to other countries [13]. Previous study revealed that 29.6% of preschool children were overweight, and 11.1% were obese with the prevalence of obesity being significantly higher for boys than girls [14]. The present study also, found increased prevalence of BMI/age Z score > 2 SD in males compared to females. In the present study, after allowing for the confounding factors, children living in the economically developed North region of Jeddah city were more likely to become obese compared to the less economically developed South region of Jeddah. In the present study, parental education or occupation were not significant predictors of BMI/age Z score over 2 SD in the studied children. These findings are supported by a previous study [4]. In the present study, gender and age of the preschool children were significantly associated with having BMI/age Z score > 2 SD, while other variables like family size, ranking of the child, smoking habits of the parents, birth weight, feeding of the child during infancy and dietary habits were not significant. In a meta-analysis study, it was found that duration of breastfeeding was inversely and linearly associated with the risk of overweight. The risk of overweight was reduced by 4% for each month of breastfeeding [15]. Nevertheless, in the current study, after allowing for confounding factors, we got contradicting results, where prolonged breast feeding was associated with increased BMI Z score among Saudi children. Previous studies found significant link between sugary drink consumption and weight gain in children [16, 17]. However, the present study did not find daily consumption of sweet food, intake of milk or fruits and vegetables as significant predictors of BMI/age Z score > 2 SD.

In the present study, although, asthma was positively associated with increased BMI Z score, this association disappeared when logistic regression was used to allow for the effects of different socio-demographic and clinical factors. Findings of the present research were supported by other study [18]. Although, previous study claimed positive association between obesity and both allergic rhinitis and eczema [19], yet the present study, failed to find such an association. Stunting is prevalent in many developing countries; it is a possible risk factor for being overweight, as it may cause a series of long-lasting changes, such as reduced energy expenditure, increased risk to the effects of a high-fat diet, reduced-fat oxidation, and impaired regulation of food intake [20]. In the present study it was found that increased BMI/Age Z Score > 2 SD was significantly associated with the stunting in preschool children.

In conclusion, the present work gives insight on the importance of increased BMI/Age in preschool children. This study demonstrated that increased BMI/Age in children can be affected by age, and socio-economic status, among other factors. The double malnutrition problem, including stunting and obesity, among preschool age children is common. Tackling increased BMI in preschool children is vital to reverse the pediatric obesity epidemic.

## Limitations of this study

The limitation of this study, was that we relied, on the recall of the clinical history of the child by the mother. However, our results were very close to studies conducted in Saudi Arabia and in other parts of the world.

## Data Availability

Authors present the data on the main paper. The raw data is available at Ibn Sina National College for medical studies, Jeddah, KSA (researchcenter@ibnsina.edu.sa).

## Abbreviations

BMI: Body mass index
SD: Standard deviation
SPSS: Statistical package for Social Sciences
Od: Odds ratio
CI: Confidence interval
Z: SD under the normal probability distribution curve
